# Twitter Analysis of Health Care Workers’ Sentiment and Discourse Regarding Post–COVID-19 Condition in Children and Young People: Mixed Methods Study

**DOI:** 10.2196/50139

**Published:** 2024-04-17

**Authors:** Macarena Chepo, Sam Martin, Noémie Déom, Ahmad Firas Khalid, Cecilia Vindrola-Padros

**Affiliations:** 1 School of Nursing Universidad Andrés Bello Santiago Chile; 2 Department of Targeted Intervention University College London London United Kingdom; 3 Oxford Vaccine Group Churchill Hospital University of Oxford Oxford United Kingdom; 4 Canadian Institutes of Health Research Health System Impact Fellowship Centre for Implementation Research Ottawa Hospital Research Institute Otawa, ON Canada

**Keywords:** COVID-19, postacute sequelae of SARS-CoV-2, PASC, post–COVID-19 condition, children, vaccines, social media, social network analysis, Twitter

## Abstract

**Background:**

The COVID-19 pandemic has had a significant global impact, with millions of cases and deaths. Research highlights the persistence of symptoms over time (post–COVID-19 condition), a situation of particular concern in children and young people with symptoms. Social media such as Twitter (subsequently rebranded as X) could provide valuable information on the impact of the post–COVID-19 condition on this demographic.

**Objective:**

With a social media analysis of the discourse surrounding the prevalence of post–COVID-19 condition in children and young people, we aimed to explore the perceptions of health care workers (HCWs) concerning post–COVID-19 condition in children and young people in the United Kingdom between January 2021 and January 2022. This will allow us to contribute to the emerging knowledge on post–COVID-19 condition and identify critical areas and future directions for researchers and policy makers.

**Methods:**

From a pragmatic paradigm, we used a mixed methods approach. Through discourse, keyword, sentiment, and image analyses, using Pulsar and InfraNodus, we analyzed the discourse about the experience of post–COVID-19 condition in children and young people in the United Kingdom shared on Twitter between January 1, 2021, and January 31, 2022, from a sample of HCWs with Twitter accounts whose biography identifies them as HCWs.

**Results:**

We obtained 300,000 tweets, out of which (after filtering for relevant tweets) we performed an in-depth qualitative sample analysis of 2588 tweets. The HCWs were responsive to announcements issued by the authorities regarding the management of the COVID-19 pandemic in the United Kingdom. The most frequent sentiment expressed was negative. The main themes were uncertainty about the future, policies and regulations, managing and addressing the COVID-19 pandemic and post–COVID-19 condition in children and young people, vaccination, using Twitter to share scientific literature and management strategies, and clinical and personal experiences.

**Conclusions:**

The perceptions described on Twitter by HCWs concerning the presence of the post–COVID-19 condition in children and young people appear to be a relevant and timely issue and responsive to the declarations and guidelines issued by health authorities over time. We recommend further support and training strategies for health workers and school staff regarding the manifestations and treatment of children and young people with post–COVID-19 condition.

## Introduction

### Background

More than 3 years after the outbreak of the COVID-19 pandemic [1], the social, political, and economic impact of this phenomenon has been more than significant, considering >700 million worldwide cases and nearly 7 million people’s deaths [2]. Given the scale of the phenomenon, it is imperative for all countries to thoroughly examine the lessons gleaned from the pandemic, particularly regarding a matter that has raised significant concern among the populace: the long-term effects experienced by individuals who have had COVID-19, spanning weeks, months, or even years after their initial infection [3]. This phenomenon, referred to as post–COVID-19 condition (or more commonly “long COVID”), warrants careful consideration and analysis [4].

There is increasing information regarding the clinical manifestation of this condition, particularly in the adult population. The worldwide prevalence has been estimated at approximately 50% to 70% in individuals hospitalized during acute COVID-19 infection and 10% to 12% in vaccinated cases [5]. While children and young people have a low likelihood of severe COVID-19 infection [6], the information available to date indicates that the presence of post–COVID-19 condition in this group may be as disabling as in adults, reaching a prevalence rate of 23.4% (range 3.7%-66.5%) [7].

An agreed definition by the World Health Organization indicates that post–COVID-19 condition in children and young people is a condition that occurs “in individuals with a history of confirmed or probable SARS-CoV-2 infection when experiencing symptoms lasting at least two months which initially occurred within three months of acute COVID-19” [8]. Post–COVID-19 condition strongly impacts daily functioning and can develop or continue after COVID-19 infection and may fluctuate or relapse over time [4,8,9].

Among the symptoms most frequently attributable to post–COVID-19 condition in children and young people are fatigue, altered smell or anosmia, and anxiety [8]. However, other symptoms have also been reported, such as sleep disturbances, difficulty in concentrating, abdominal pain, myalgia or arthralgia, earache or ringing in ears, mood swings, persistent chest pain, stomach pain, light sensitivity, diarrhea, heart palpitations, and skin lesions [8,10]. One of England’s most significant studies is the Children and Young People With Long COVID study by Stephenson et al [11]. This national research matched longitudinal and cohort studies in adolescent individuals aged 11 to 17 years and found the presence of symptoms in 35.4% of the adolescent individuals who tested positive at baseline and 8.3% who of the adolescent individuals who tested negative at baseline. A total of 3 months after testing, 66.5% of those who tested positive and 53.3% of those who tested negative had any symptoms [11]. However, Stephenson et al [12] recently indicated that in a 6-month follow-up, the prevalence of specific symptoms reported at the time of the polymerase chain reaction testing decreased over time, where, for example, the prevalence of chills, fever, myalgia, cough, and sore throat among those who tested positive decreased from 10% to 25% to <3%.

As research on the symptoms, prevalence, and treatment of post–COVID-19 condition in children and young people continues, it is essential to add to the literature by developing studies that determine the condition’s impact on this group, considering that they are experiencing a range of unwanted symptoms that disrupt their quality of life and that of their families.

Considering that listening to the voices of families and health workers could be helpful to broaden the knowledge achieved in post–COVID-19 condition in children and young people, a powerful tool could be social media, such as Twitter (subsequently rebranded as X). With >3729 million daily active users, Twitter has become one of the most important social platforms in the world [13]. People used Twitter during the COVID-19 pandemic for different purposes, such as world leaders communicating with citizens [14,15], organizations monitoring movement [16], scientists studying public discourse around the pandemic [17,18], and researchers performing sentiment analysis [19-21]. In the case of physicians and health care workers (HCWs), Twitter has been used to share and evaluate scientific evidence, guidelines, and technical advice [22-24] and track the course and burden of disease [25].

### Objectives

Using the social media monitoring platform Pulsar [26], we aimed to explore HCWs’ perceptions concerning post–COVID condition in children and young people in the United Kingdom between January 2021 and January 2022. We aimed to contribute to the emerging knowledge on post–COVID-19 condition in children and young people and identify critical areas and future directions for researchers and policy makers.

## Methods

### Overview

We considered a mixed methods approach to be a pragmatic research paradigm. We analyzed data by conducting a Collaborative and Digital Analysis of Big Qualitative Data in Time Sensitive Contexts (LISTEN) [27]. This mixed methods analysis consisted of iterative cycles intercalating team discussion and using digital text and discourse analytics tools to analyze related social media data [27]. We used the LISTEN method to perform quantitative and qualitative analyses of Twitter posts, extracted through the Pulsar platform [26], related to the experience of post–COVID-19 condition in children and young people in the United Kingdom (eg, phrases, words, hashtags, videos, and images), published between January 1, 2021, and January 31, 2022. We created an advanced Boolean search for keywords mentioning “long COVID” and corelated words, hashtags, and symptoms; furthermore, we filtered for user accounts who identified as HCWs in their Twitter biography description (Multimedia Appendix 1).

Quantitative analysis of all tweets included the following: (1) engagement analysis, where we specifically measured reactions to posts, for example, a retweet, a share, or a comment or quote made toward a tweet; (2) sentiment and emotion analysis, where we measured the positive or negative sentiment in the words and tone of each post within the context of post–COVID-19 condition and HCW’s roles (Multimedia Appendix 2); (3) emotion analysis, where we measured the emotions expressed in the tweets, classified as sadness, anger, disgust, fear, and joy; (4) frequency analysis, where we observed the frequency of keywords and themes in the data set; (5) segmentation analysis, where we measured the key connections or relationships between keywords and their frequent use in the same context; (6) demographic analysis, where we measured the occupation, gender (man or woman or nonbinary or unknown), and city of origin related to the users posting tweets; and (7) analyses, where we evaluated the most influential accounts and the most mentioned websites.

Big qualitative analysis was carried out through thematic discourse analysis of the data sample, using InfraNodus [28], specifically analyzing the key themes and topics of concern expressed throughout the data set. A codebook was constructed based on the mapping of themes agreed upon by 3 researchers (ND, SM, and MC; Multimedia Appendix 3).

The principal investigators (ND, AFK, SM, and MC) interpreted and analyzed the data collected, following the recommendations for rigorous research provided by Creswell and Poth [29]. Using the LISTEN method [27], we aimed to show that the integration of qualitative insights through thematic analysis with the quantitative backing of topic modeling can offer a comprehensive view of the discourse. This mixed methods approach allows us to capture the richness of qualitative data while leveraging the objectivity of quantitative measures. Our initial data harvest of the larger corpus data from the Pulsar platform captured 300,000 tweets; this data harvest helped to underpin the software’s sentiment analysis modeling of this specific data set, providing a robust quantitative foundation. The addition of further qualitative data analyses from a smaller qualitative sample allowed for an in-depth understanding of nuanced conversations, particularly when exploring new or complex phenomena such as post–COVID-19 condition in children and young people, with the provision of insights into the context, subtext, and sentiment behind the tweets offering valuable snapshots of public perception and discourse. We used an iterative mixed methods approach, iterating between team discussions and using digital analytics tools to discern relevant themes from the Twitter data corpus. Specifically, we used InfraNodus for thematic analysis, which incorporates a topic modeling script for analyzing and identifying key topics of concern with a data set and provides a structured and objective interpretation of the data. The coding process involved 3 independent researchers (MC, SM, and ND), each with expertise in health care, social network analysis, and digital global health. When initial coding disagreements arose, we meticulously tagged any queries and discussed the posts in question. These instances led to 3 structured meetings wherein the research team deliberated collaboratively to resolve conflicting interpretations. This approach resulted in an 81.99% (2122/2588) initial intercoder agreement rate for the tweets analyzed. For the remaining instances where consensus was not initially reached, the majority rule was applied to finalize theme codings. To quantify the reliability of our coding procedure, with 81.99% (2122/2588) of the tweets coded identically, we used the Cohen κ score, which provides a measure of interrater agreement adjusted for chance. Including the calculation of all variations, this score was calculated to be approximately κ=0.70, indicating good agreement among the coders.

### Ethical Considerations

The study only collected data from publicly accessible social networks that have been anonymized by various means, particularly by replacing all usernames and links with anonymous text and summaries of tweets that have been edited, retaining the original message, avoiding direct quotations being identifiable, and ensuring that no information is provided on the identity of the individuals who posted the content studied on the platform.

Internet research requires researchers to carefully consider guidelines to determine whether ethics approval and informed consent are needed [30]. On the basis of the terms set out by the Research Ethics Committee at the University College London [31], the study was considered exempt from formal ethics approval for the following reasons: (1) study involving information freely available in the public domain, such as published biographies, newspaper accounts of an individual’s activities, and published minutes of a meeting, that although is considered personal under the Data Protection Act, would not require ethics review; and (2) study involving anonymized records and data sets in the public domain, such as data sets available through the Office for National Statistics or the UK Data Archive where appropriate permissions have already been obtained and it is not possible to identify individuals from the information provided.

Therefore, we anonymized all records and data sets collected during the study to make identification impossible. We removed social media usernames from the data samples. No direct or easily traceable quotes have been included. These measures align with best practices [32-35]. While this study was beyond the scope of the human ethics committee, we adhered to the principles of ethics: beneficence, nonmaleficence, autonomy, and justice [36]. We collected and analyzed data through secure encrypted servers via the Meltwater and InfraNodus platforms.

## Results

### Audience Analysis

During the period from January 2021 to January 2022, we obtained 300,000 tweets from 936 accounts. After filtering for relevant posts (refer to inclusion and exclusion criteria in Multimedia Appendix 1), we analyzed a sample of 2588 tweets using mixed methods analysis. In terms of gender (man, woman, nonbinary, or unknown), 32.88% (851/2588) were female individuals, 23.49% (608/2588) were male individuals, and 43.59% (1128/2588) were unknown. According to the description given in the user’s biography, the most frequently self-reported terms were “NHS” (582/2588, 22.49%), “health” (230/2588, 8.89%), “medical” (168/2588, 6.49%), “nurse” (166/2588, 6.41%), “clinical” (160/2588, 6.18%), “mum” (158/2588, 6.11%), “doctor” (145/2588, 5.6%), and “GP” (145/2588, 5.6%). In terms of city, tweets came mainly from London (958/2588, 37.02%), Newcastle upon Tyne (326/2588, 12.6%), Redcar (160/2588, 6.18%), Manchester (140/2588, 5.41%), and Bradford (111/2588, 4.29%).

### Profession

Regarding profession described in the user’s biography, the most frequently mentioned roles were nurses (176/2588, 6.8%); medical roles, for example, paramedic and nursing assistant (173/2588, 6.68%); clinical roles, for example, surgeon, physiotherapist, and anesthesiologist (160/2588, 6.18%); general practitioners (GPs), for example, hospital GP or local surgery GP (142/2588, 5.49%); and physician (140/2588, 5.41%). The most frequent organization affiliated with was the National Health Service (587/2588, 22.68%).

### Most Influential Accounts

One of the accounts that generated the highest number of mentions and, therefore, some of the most influence, as they were the ones that talked the most about post–COVID-19 condition in children and young people, was the account for @longcovidkids (593/2588, 22.91% tweets), related to the most shared website *longcovidkids.org* [37]*,* an international UK-based charity for families and children living with post–COVID-19 condition. Although the account was created in October 2020, it was first mentioned in our data collection timeline on January 1, 2021. It offers web support services, funding, and research participation and represents children and young people living with post–COVID-19 condition in expert forums, research panels, health organizations, and parliamentary groups. The other most shared web pages were theguardian.com (the United Kingdom) [38], bbc.co.uk (the United Kingdom) [39], peoplewith.com (the United States) [40], and ncbi.nlm.nih.gov (the United States) [41]. This shows that in the United Kingdom, there was a mixed influence of UK and US link resources linked to HCW Twitter users in the United Kingdom.

### Keyword Analysis

The volume of social media engagement in the discussion about the post–COVID-19 condition experience in children and young people in the United Kingdom reached 1400 posts, 1550 engagements, and 1.9 million impressions. Overall, comments were very responsive to government decisions regarding the vaccination program and school closures (Multimedia Appendix 4). During the first peak of comments in January 2021, the amount of discourse expanded leading up to March 2021, when there were different announcements of school closures, and the guidelines were delivered regarding the priority groups of the vaccination program (frontline HCW and people aged >80 years first). The highest engagement was between June and July 2021, which coincides with the government announcement regarding the availability of vaccines for people aged >18 years. The third peak of comments occurred in September 2021, the same month the authorities announced the extension of the vaccination program to children aged 12 to 15 years.

### Top Keywords Analysis

The top words in posts associated with children and young people’s experience of post–COVID-19 condition in the United Kingdom were “Children” (352/2588, 13.6%), “kids” (160/2588, 6.18%), “people” (158/2588, 6.11%), “Young” (148/2588, 5.72%), and “schools” (83/2588, 3.21%). The top hashtags were #longcovid (1387/2588, 53.59%), #longcovidkids (448/2588, 17.31%), #covid19 (370/2588, 14.3%), and #covid (176/2588, 6.8%).

### Sentiment and Emotions Analysis

According to sentiment analysis, 99.38% (2572/2588) of the posts reflected negative sentiments and 0.62% (16/2588) reflected positive sentiments. Negative sentiments were mainly associated with comments on hospitalization figures related to the COVID-19 pandemic, criticism of pandemic mitigation policies, and vaccination of children and young people. Furthermore, positive sentiments mainly concerned acknowledgments around decreasing numbers of community support groups.

The primary emotions identified were as follows:

- Sadness (1752/2588, 67.7%), such as in the following tweet:

@[Username] Really upset, after my tough on-call last night. Hospitalisations are still going up, and Gov announcement minismises the effect of long-COVID in adults and children. It’s so hard to keep spirits up today. But we’ll try and continue doing our best in the NHS.

- Joy (367/2588, 14.18%), such as in the following tweet:

@[Username] It’s been an amazing day! [...] I’ve been able to share the experience I’ve gained treating children and adolescents with Long COVID over the last year.

- Fear (233/2588, 9%), as seen in the following tweet:

@[Username] It’s really urgent that young people get the message that they need to get vaccinated. Long COVID is ruining many people’s lives! It’s not a lie or hypochondria, there are real, physiological changes, please understand!

### Segmentation Analysis

This analysis revealed the critical clusters of conversation around the main topics of concern within the discourse network around post–COVID-19 condition. Comments were distributed in 4 key conversation segments as follows:

1. People, schools, and prevention (1734/2588, 67%): Most of the comments related to measures taken in terms of COVID-19 prevention in schools, concern about the risk of exposure, and sharing experiences of infection in schools.
2. Health, adults, and impact (401/2588, 15.49%): Comments mainly reflected concerns and uncertainty about the long-term effect of post–COVID-19 condition on both children and young people and adults.
3. Cases, virus, and risk (326/2588, 12.6%): Comments reflected worries about the associated risks and long-term consequences attributable to post–COVID-19 condition (in both adults and children and young people) and the constant mutation of the virus, which will create a permanent risk in the population.
4. Months, distress, and symptoms (106/2588, 4.1%): Some HCWs used Twitter to share how children and young people experience post–COVID-19 condition and the extent of these symptoms. Some HCWs exemplified certain typical manifestations, such as fatigue.

### Discourse Analysis by Theme

**Figure 1:**
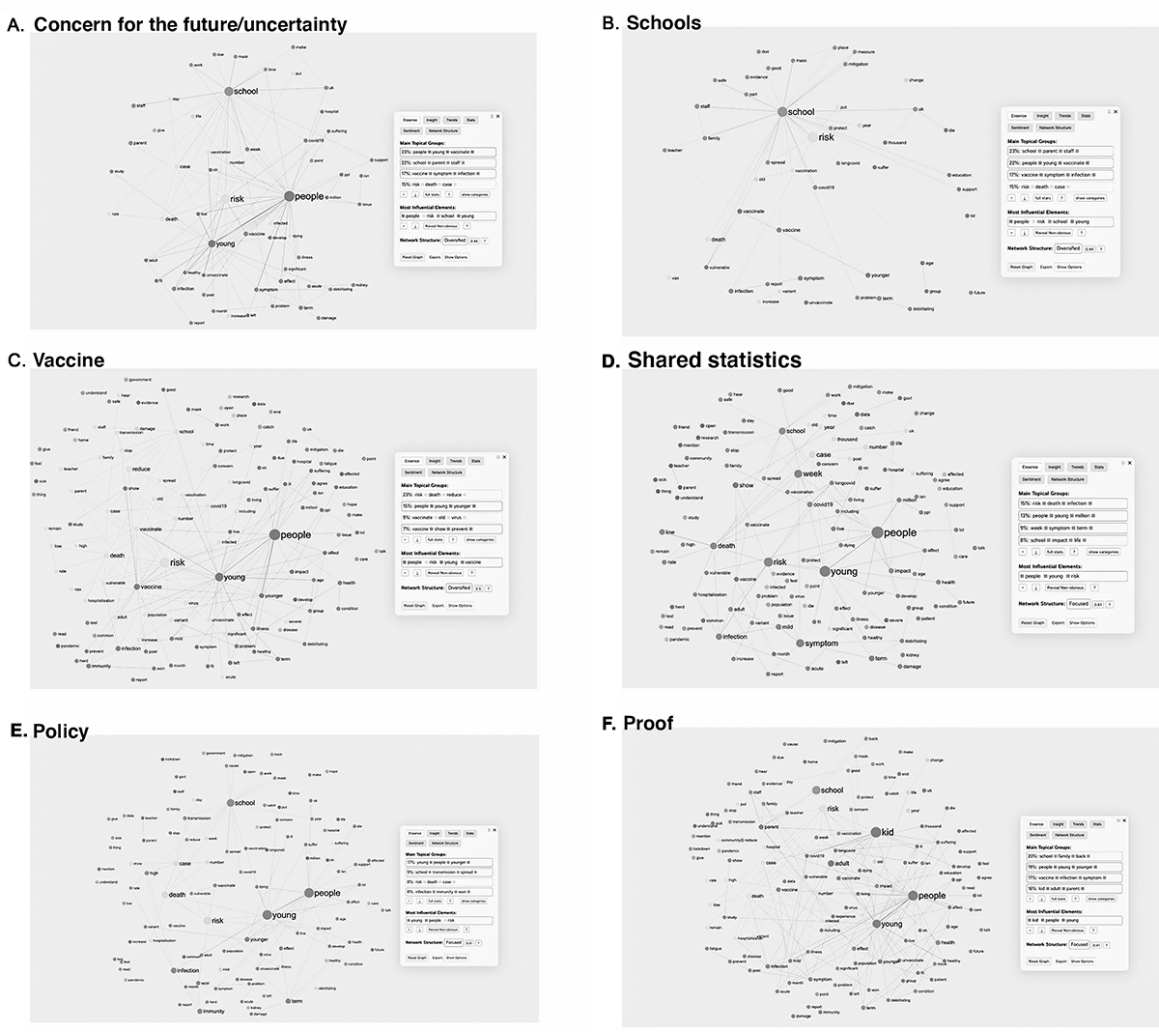
Discourse analysis by theme: (A) concern for the future or uncertainty, (B) schools, (C) vaccine, (D) shared statistics, (E) policy, and (F) proof.

**Figure 2:**
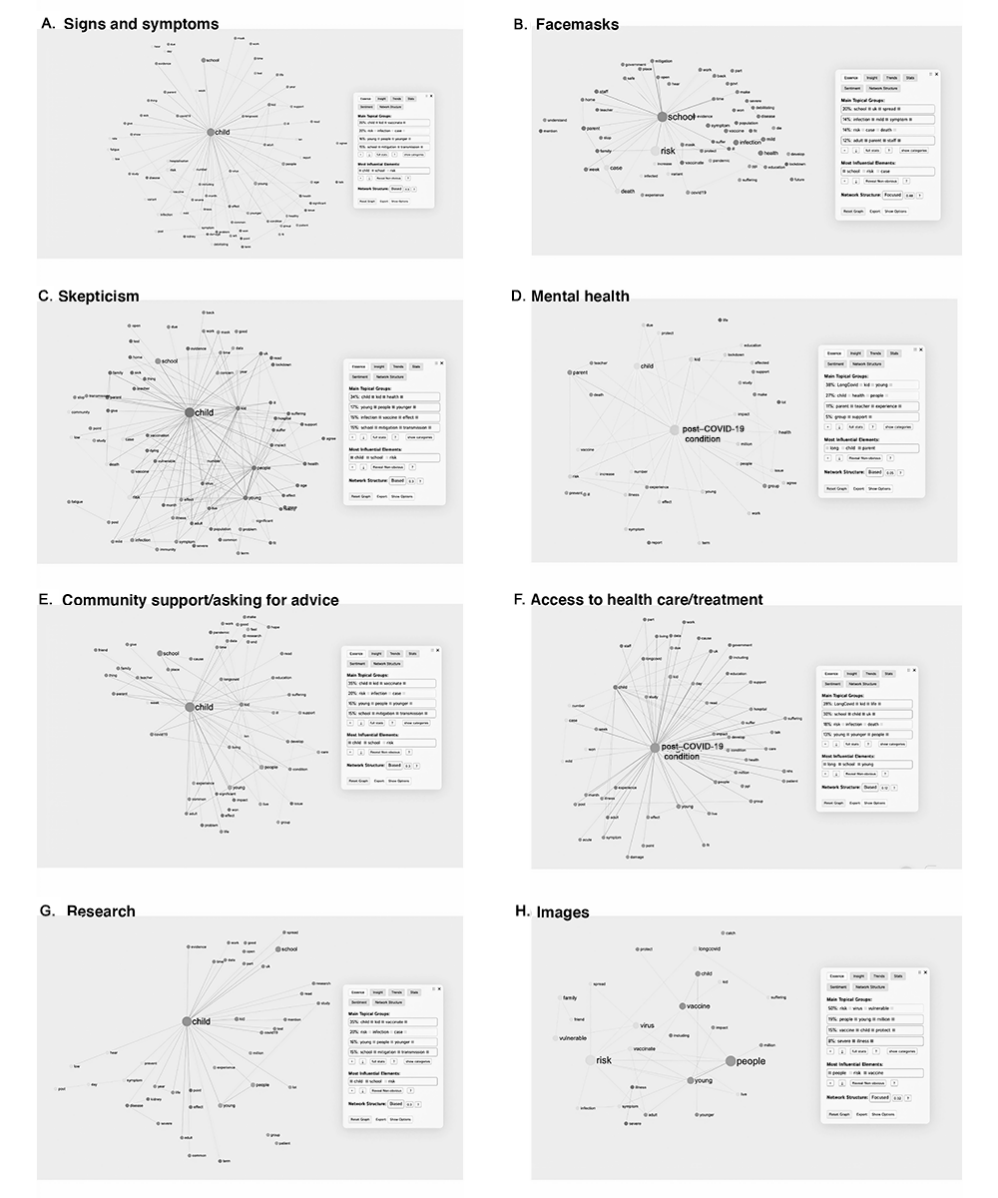
Discourse analysis by theme: (A) signs and symptoms, (B) face masks, (C) skepticism, (D) mental health, (E) community support or asking for advice, (F) access to health care or treatment, (G) research, and (H) images.

To better understand the topics discussed from the segmentation analysis, we performed a discourse analysis of the key co-occurring themes and topics of concern shared within discussions regarding post–COVID-19 condition in children and young people. The following themes emerged (Textbox 1): concern or uncertainty for the future, school attendance, mask protection from COVID-19, vaccine uptake, infection rates, policy (support or skepticism), understanding and visualizing symptoms, child mental health, access to care, community support, and research (Figures 1 and 2).

**Discourse analysis themes.**

- Concern for the future or uncertainty (615/2588, 23.76% tweets): Most comments showed a concern for the future, focusing on shared statistics regarding the rate and spread of infection in children and young people and how this would affect future health outcomes. Furthermore, this group expressed concern regarding political decisions; the presence of illness in loved ones; the eventual overload and response capacity of the health system in the face of an increase in post–COVID-19 condition cases; and the need for training of health care workers (HCWs) to deal with comorbid, potentially long-term symptoms (Figure 1A).
- Schools (460/2588, 17.77% tweets): Comments aimed to promote vaccination policies for schoolchildren and flexible measures regarding teachers’ work and attendance, considering cases of people with prolonged symptoms. In addition, several tweets expressed dissatisfaction with school risk mitigation measures, such as the use of face masks and air filters (Figure 1B).
- Vaccine (386/2588, 14.9% tweets): Most tweets from this group showed their disapproval of the constant changes in the government’s decisions regarding schools and priority groups for vaccination. Between March and June 2021, the first set of tweets criticized the lack of priority in the vaccination program for schoolchildren and other at-risk groups (such as teachers). Once the authorities announced a vaccination program for schoolchildren aged 12 to 15 years (Multimedia Appendix 4), most comments promoted vaccination for this group. A few comments (78/2588, 3.01%) shared concerns about the vaccine’s efficacy for children, based on the experiences of COVID-19 reinfection in adults despite having received the recommended initial doses. However, to a lesser extent (26/2588, 1%), there was a refusal to vaccinate children, citing fear of possible adverse effects. Nonetheless, it is worth noting that the community frequently refuted such comments (Figure 1C).
- Share statistics (334/2588, 12.91% tweets): Frequently, HCWs shared statistical data, such as the number of affected children and young people, the number of post–COVID-19 condition cases, and hospital admissions and deaths. Some of these data were used to validate the existence of the post–COVID-19 phenomenon or to express concern about it (Figure 1D).
- Policy (316/2588, 12.21% tweets): The comments were responsive to the policies emanating from the authorities over time (Multimedia Appendix 4). There were 5 main criticisms, including changes in school closure or opening policies; HCWs question why the authorities ignore the evidence of post–COVID-19 cases in children and young people, leading them to question whether decision makers have sufficient training to control the pandemic adequately; the failure to include teachers and school workers in the COVID-19 vaccination program as well as the younger population; the lack of mitigation measures in schools, such as improvements in ventilation systems and mandatory use of masks; and the herd immunity as a plan in the government’s *hidden agenda*, that is, to promote work and activate the economy (Figure 1E).
- “Proof” (280/2588, 10.82% tweets): Most tweets in this group argued regarding the existence of children and young people with post–COVID-19 condition through pictures; statistics; scientific papers; and personal, family, and professional experiences (Figure 1F).
- Signs and symptoms (189/2588, 7.3% tweets): Among the symptoms described, chronic fatigue and exhaustion were the most frequent symptoms, which prevent normal activities. Other symptoms were respiratory: dyspnea, chronic cough, and shortness of breath; gastrointestinal: acute or intense abdominal pain, nausea, bloating, gastroparesis, and change in smell or taste; muscular: severe joint pain, “painful foot” and difficulty with physical activity; mental health: anxiety and low mood; topical: rash, skin rashes, and redness and pain in the eyes; and nonspecific symptoms, such as chest pain, heart palpitations, constant high body temperature, precocious puberty, hormonal changes, and erectile dysfunction (Figure 2A).
- Face masks (119/2588, 4.6% tweets): Face masks were widely promoted, especially in schools, because HCWs considered them as a practical and straightforward strategy to control the pandemic (Figure 2B).
- Skepticism (101/2588, 3.9% tweets): Comments showed reticence toward post–COVID-19 condition in children and young people. Some of the arguments focused on a perceived lack of clarity in the clinical manifestations and stressed the need to better differentiate the post–COVID-19 condition from other related symptomatologies, such as mood disorders (eg, depression and anxiety due to confinement). In contrast, several arguments agreed on the need for more scientific evidence, arguing that post–COVID-19 condition in children and young people are isolated. Other users claimed not to know of such cases instead of calling post–COVID-19 condition in children and young people SMS text message an exaggeration. In addition, several arguments favored releasing restrictions for children and young people, particularly arguments related to the use of masks, because of possible associated risks, for example, hypoxia (Figure 2C).
- Mental health (54/2588, 2.09% tweets): Symptoms attributable to mental health problems in children and young people were also a concern. For instance, HCWs mentioned sadness, fear of infecting their family, anxiety regarding sick parents, stress, night terrors, self-harm, and suicidal ideation. Furthermore, users discussed a perceived lack of specific support for children and young people and their families in situations such as hospitalization; prolonged COVID-19 condition; admission to intensive care; and death of a family member, schoolmate, or teacher, all situations that triggered permanent stress in these groups (Figure 2D).
- Community support or asking for advice (93/2588, 3.59% tweets): Some HCWs used Twitter to ask for guidance on a specific issue or share experiences of having post–COVID-19 condition or caring for children and young people or family members. Furthermore, they shared informative infographics provided by experts regarding post–COVID-19 condition in children and young people (Figure 2E).
- Access to health care or treatment (72/2588, 2.78% tweets): Some HCWs mentioned the lack of specialist (cardiology) support, concerns regarding prolonged National Health Service burnout, and criticisms regarding how follow-up was carried out concerning the relative symptomatology of children and young people with post–COVID-19 condition. At the same time, opening new centers for children and young people with post–COVID-19 condition generated different reactions. On the one hand, some HCWs recognized it as a substantial development, but on the other hand, some HCWs recognized it as proof of the existence of post–COVID-19 condition in children and young people, which raised concerns for the future (Figure 2F).
- Research (52/2588, 2% tweets): Under this theme, tweets largely promoted study on post–COVID-19 condition in children and young people or highlighted the need for further study on the subject (Figure 2G).
- Images (57/2588, 2.2% tweets): Images shared were primarily from scientific studies, including infographics (from organizations such as National Health Service or @LongCovidKids) and visualization of children and young people’s symptoms, such as rashes, COVID-19 toe, and joint pain. Most infographics shared by organizations (and not individuals), such as the organization LongCovidKids, were related to statistics, such as the number of children and young people with post–COVID-19 condition or the quantification of the type of symptoms experienced. Shared photographs tended to show the more “visually recognizable” symptoms of post–COVID-19 condition, such as skin lesions, rashes, or inflammation. The less visible symptoms, such as chronic fatigue and neurological issues, were represented with photographs of children and young people lying, sleeping under blankets, or duvets or on hospital beds (Figure 2H).

## Discussion

### Principal Findings

Our primary objective was to explore HCWs’ perceptions concerning post–COVID-19 condition in children and young people in the United Kingdom between January 2021 and January 2022. Our findings indicated that comments made by HCWs on Twitter were responsive to announcements issued by authorities regarding the management of the COVID-19 pandemic in the United Kingdom and associated regulations on the operation of schools. The most frequent feelings and emotions were negative, mainly sadness. In turn, we identified relevant themes for HCWs, such as uncertainty or concern about the future; policies; and regulations for the prevention, management, and addressing both COVID-19 and post–COVID-19 condition in children and young people; vaccination; and the use of Twitter as a strategy to share scientific literature, management strategies, and clinical and personal experiences.

Concern from HCWs regarding the policies for addressing the COVID-19 pandemic in the children and young people in the United Kingdom (including vaccination and schools) was a recurring theme in our findings. Furthermore, concern regarding the side effects of the COVID-19 vaccine and how the vaccine might interact with preexisting physiological symptoms of post–COVID-19 condition in children and young people was a topic of discussion. Similarly, the constant change in policy making in the United Kingdom, as public health bodies and governments have tried to understand and adapt to the emergence of post–COVID-19 condition, have added to the strength of this ongoing debate [42]. The lack of up-to-date evidence on post–COVID-19 condition in children and young people prompted HCWs to rely on Twitter during the pandemic to communicate relevant information. Twitter has a broad audience reach; is used as a communication tool by politicians, health bodies, and other key influences; and facilitates real-time updates [43]. During the pandemic, HCWs, primarily those in frontline roles and local response coordination, have often been challenged to become credible spokespersons for pandemic information [44]. Such credibility directly influences public confidence and decision-making, ultimately determining the success or failure of a public health intervention [43].

Furthermore, failures in risk communication could explain the presence of uncertainty and negative feelings associated with school regulations. When people are upset, distressed, or fearful, they often do not trust the authority, decrease the perceived validity of the communication received, and find information processing difficult [45]. In this regard, Fotheringham et al [46] indicated that during 2020, school leaders in the United Kingdom faced pressures and challenges related to translating and enacting school policies, particularly with the perceived lack of agency shared by the government concerning being able to translate centrally issued guidelines. In turn, Tomson et al [47] reported that the pandemic has negatively impacted the well-being of leaders in all types of schools and across all demographic groups, affecting their ability to think clearly and solve work-related problems. Given that the protection and care of children and young people health during the COVID-19 pandemic ultimately rests with school leaders, the search for support strategies that focus on the needs of these groups becomes an urgent necessity.

### Findings in Relation to Other Studies

Using Twitter’s information, this is one of the first studies to capture health professionals’ perceptions of prolonged COVID-19 in the children and young people in the United Kingdom. However, other studies have addressed post–COVID-19 condition on this social network. Callard and Peregov [48] reviewed how, through social platforms such as Twitter, patients made the persistence and heterogeneity of COVID-19 symptoms visible, thus catapulting the inclusion of post–COVID-19 condition as a relevant phenomenon in clinical and policy debates. In contrast, other authors in the last 2 years have explored on various platforms (including Twitter) the persistence of symptoms and emotional impact after months of suspected and confirmed diagnosis of COVID-19 [49-55], including the period of vaccination. Furthermore, others have explored web discussions regarding this phenomenon [56]. Several of these authors agree on a perceived lack of support and specific resources from governmental bodies, a lack of information or clarity in the instructions given, and the absence of formal mechanisms to allow the voices of patients and the community to be heard. The above point is critical as it highlights the gap between the needs of the population and the response provided by policy makers, which not only translates into a gap in access to health services but also limits citizen participation in decision-making on the issues that affect their own health and increases distrust toward regulations and instructions issued by the government.

### Implications for Policy and Practice

#### Overview

Several policy recommendations and implications are targeted at various stakeholders to consider while implementing future policy guidelines to address post–COVID-19 health care delivery. First, policy makers should consider investing appropriate resources to collect data regarding post–COVID-19 condition in children and young people, specifically on the impact of COVID-19 on the mental health of children and young people. This implies working closely with researchers to streamline data collection and reporting on post–COVID-19 condition. Second, policy makers should consider providing a basic level of psychosocial support with access to quality mental health and psychosocial support services for HCWs, school staff, parents, and children and young people experiencing post–COVID-19 condition. This implies strengthening health systems, community-based programming, and mobilization. Policies must include documenting the impact of mental health and psychosocial support interventions and innovative approaches to be more widely disseminated and scaled up across different contexts and target population groups. Third, to address the criticism around frequent changes in school closure and opening policies, decision makers should develop clear, easy-to-understand school mitigation plans informed by the best available evidence. The plans should incorporate teachers, school workers, and parents to ensure all voices are included in the policy plan. Fourth, policy makers should adopt a shared decision-making approach incorporating HCWs in the decision-making process for managing the COVID-19 pandemic. Finally, government decision makers should set post–COVID-19 pandemic recovery policies informed from a health equity perspective and how this affects children and young people living with post–COVID-19 condition, factoring in childhood, family income, housing, domestic violence, access to health care, and racism.

In terms of the needed clearer road map for recommendations to support training strategies for HCWs and school staff regarding post–COVID-19 condition in children and young people, we have outlined the following 10 steps.

#### Step 1: Data Collection and Analysis

Our study underlines the critical need for comprehensive data on post–COVID-19 condition’s impact on the mental health of children and young people. As a first step, it is recommended that policy makers should allocate resources for the systematic collection and analysis of data on post–COVID-19 condition in children and young people, particularly focusing on mental health outcomes. These data should be used to identify the most prevalent symptoms and the most effective treatment strategies. In this context, it is recommended that experts emphasize the importance of early detection and medical consultation for mental health issues in children and young people diagnosed with post–COVID-19 condition, including mood changes, irritability, social withdrawal, memory problems, difficulty in concentrating, anxiety, depression, posttraumatic stress, school absenteeism, and suicidal ideation [57,58]. This entails working closely with researchers to streamline data collection and reporting on post–COVID-19 condition.

#### Step 2: Psychosocial Support Framework

It has been noted that globally, programs for managing post–COVID-19 condition in children and young people are heterogeneous, ranging from the use of physiotherapy, pediatric occupational therapy, and psychological support to interventions aimed at lifestyle modifications [59]. This diversity could impact differential outcomes in the treatment, recovery, and timely and effective rehabilitation of children and young people with post–COVID-19 condition. Upon analyzing the wider literature and the social media data in this study, it is recommended that a basic level of psychosocial support should be established. This would involve ensuring access to quality mental health services for HCWs, school staff, parents, and children and young people with post–COVID-19 condition. This framework should be integrated into the health system and community-based programming, emphasizing the mobilization of resources and strengthening of support networks. It is suggested that the psychosocial support framework should facilitate access to quality mental health services and support networks that are robust and responsive. Community engagement gleaned from further Twitter discourse analysis should be a helpful guide in the development of these services to ensure they meet the real and expressed needs of children and young people with post–COVID-19 condition. Practical examples of basic psychosocial support include using web support services; individual or group therapy sessions; school-based emotional support programs; and counseling sessions aimed at parents, family members, or school staff.

#### Step 3: Educational Mitigation Plans

The frequent policy changes around school closures highlight the necessity for stable and clear educational mitigation plans. It is recommended that these plans should be directly informed by the evidence collected and further analysis of sentiments and emotions surrounding post–COVID-19 condition in schools. Incorporating the viewpoints of teachers, parents, and school staff, as identified in our thematic analysis, will ensure that the mitigation strategies are comprehensive, feasible, and sensitive to the psychosocial impact on children and young people. School staff and policy makers should collaborate to develop clear, evidence-informed educational mitigation plans. These plans should be straightforward and involve teachers, school workers, and parents in their creation, ensuring a unified approach that considers the voices of all stakeholders.

#### Step 4: Shared Decision-Making in Health Care

In health care settings, the adoption of a shared decision-making model is crucial, enabling HCWs to actively contribute to the formulation of COVID-19 and post–COVID-19 policies. This inclusive approach ensures that frontline workers can provide valuable insights toward policy development. To facilitate this, the establishment of advisory committees composed of representatives from HCWs is recommended. This committee can convene regularly to deliberate on key decisions pertaining to the COVID-19 pandemic management, including prevention measures, resource distribution, and vaccination strategies. Such collaborative groups have demonstrated effectiveness in identifying priority needs within the context of a pandemic [60].

#### Step 5: Health Equity in Policy Setting

Post–COVID-19 recovery policies should be set with a health equity lens. This means considering factors such as family income, housing, domestic violence, access to health care, and racism and how these factors affect children and young people living with post–COVID-19 condition. Our findings emphasize the importance of framing post–COVID-19 recovery policies through a lens of health equity. The concerns raised by HCWs regarding the socioeconomic impacts, such as family income and access to health care, underline the need for policies that address not just the medical aspects of post–COVID-19 condition but also the social determinants of health. An equitable approach will ensure that children and young people from diverse backgrounds receive appropriate support.

#### Step 6: Documenting and Disseminating Interventions

It is vital to document the impact of mental health and psychosocial support interventions. In this context, it is crucial to implement innovative strategies to disseminate unbiased information about post–COVID-19 condition among health care professionals and educators working with children and young people, ensuring it reaches different contexts and populations. These strategies may include creating interactive multimedia resources, such as videos and mobile apps; organizing webinars; actively using social media; and forming web support groups. These groups will provide a space where patients, health care professionals, and educators can share their experiences and knowledge regarding post–COVID-19 condition. These actions will not only help reduce isolation and social stigma but also strengthen support for these groups considered vulnerable [61].

#### Step 7: Developing a Clear Communication Strategy

Policy makers must develop a clear communication strategy to address frequent policy changes and mitigate confusion. This strategy should be informed by the data collected and analysis conducted in Step 1. The data reveal a palpable sense of uncertainty and frustration due to frequent policy shifts, underscoring the need for a clear and consistent communication strategy. This strategy should be grounded in the evidence gathered from the health care community’s discourse and aim to minimize confusion by providing timely, transparent, and reliable information regarding post–COVID-19 policies and support services.

#### Step 8: Training and Support Strategies

On the basis of the findings of the comprehensive data analysis, specific training and support strategies should be developed for HCWs and school staff. These strategies should be focused on the practical aspects of identifying and managing post–COVID-19 condition in children and young people. For instance, training sessions could include practical workshops on recognizing post–COVID-19 symptoms in children and adolescents, conducting diagnostic assessments, and implementing appropriate treatment and support interventions.

#### Step 9: Continuous Feedback and Policy Adaptation

The continuous evolution of the post–COVID-19 phenomenon demands an iterative approach to policy making. On the basis of our study, we recommend establishing feedback mechanisms with HCWs and school staff to monitor the reception and effectiveness of implemented policies. This feedback, coupled with ongoing research, should inform policy adaptations to ensure they remain aligned with the evolving landscape of post–COVID-19 condition and its impact on children and young people.

#### Step 10: Evaluation and Research

Finally, there should be a commitment to ongoing evaluation and research. This will involve not only monitoring the implementation of the abovementioned steps but also supporting new research to fill any remaining gaps in understanding the long-term effects of COVID-19 on children and young people.

This sequence of steps is designed to be iterative and responsive, ensuring that the recommendations from the study are translated into concrete actions that adapt to emerging data and research findings.

### Strengths and Limitations

A key strength of this study is that our social media analysis of post–COVID-19 condition contributes toward an emerging understanding of reported experiential, emotional, and practical dimensions of post–COVID-19 condition in children and young people specifically and questions of vaccine hesitancy in children and young people with post–COVID-19 condition. This is one of the few studies to collect HCWs’ perceptions regarding post–COVID-19 condition in children and young people in the United Kingdom using information from Twitter. We identify key areas that need considering attention and focus, such as the provision of psychosocial support with access to quality mental health resources to alleviate the impact of post–COVID-19 condition in children and young people and the development of clear post–COVID-19 pandemic recovery guidelines that are informed by health equity perspective, and how this affects children and young people living with post–COVID-19 condition.

One of the limitations this study acknowledges is the definition of post–COVID-19 condition in children and young people. When data were collected, the lack of consensus on the definition of post–COVID-19 condition in children and young people forced us to formulate a definition of post–COVID-19 condition in children and young people based on the available literature. Furthermore, this study is limited to the perceptions of people who used descriptors in their web biography attributable to HCWs; therefore, our results only represent some HCWs in the United Kingdom and those in other countries. In turn, this research collected data from Twitter only; therefore, further inquiry into HCWs’ perceptions of post–COVID-19 condition in children and young people required expanding to other data sources or social networks and including languages other than English. We acknowledge that demographic factors, geographic location, and individual daily activities of social media users can significantly influence language use and word choice, introducing potential biases in tweet-based data. Such biases are inherent in any analysis of social media content and can affect the generalizability of findings. For instance, our study relies on Twitter data, which do not encompass the full spectrum of global or the UK public opinion on post–COVID-19 condition in children and young people. While Twitter serves as a valuable platform for capturing real-time sentiments and experiences, it is not fully representative of all demographics and geographic regions. Our results may reflect the perspectives of more vocal or active social media users, which may not correspond to the silent majority or those without access to social media. In addition, the absence of geotagged information for many users limits our ability to conduct a more nuanced spatial analysis of the sentiments expressed.

Furthermore, our study is built upon the recognition that social media data may overrepresent certain demographic groups while underrepresenting others, such as the older population or those without reliable internet access. This skew can influence the apparent prevalence of certain views or experiences of post–COVID-19 condition. Moreover, individuals’ patterns of daily life, reflected in their social media use and content, contribute additional layers of complexity and potential bias to the discourse analyzed.

Consistent with scholarly precedents on the subject [62,63], our study acknowledges these biases as intrinsic limitations of social media–based research. Although our analysis did not control for these factors, we recognize their potential impact on our results. Future studies would benefit from incorporating a broader array of data sources, including interviews or focus groups, to provide a more representative and comprehensive understanding of post–COVID-19 condition in children and young people. This approach would complement our Twitter-based findings and help mitigate the biases inherent in social media data.

### Conclusions

More than a year after the start of the COVID-19 pandemic, the perceptions described on Twitter by HCWs concerning the presence of post–COVID-19 condition in children and young people appear to be a relevant and timely issue as well as very responsive to the declarations and guidelines issued by the health authorities over time. The most prominent group within the discourse studied was the activist or lobbying organization @LongCovidKids, which shared the most tweets and images over the period studied. We recommend that future research focus on how web health activism is organized and carried out for children and young people with post–COVID-19 condition. Such a strategy would allow for a better understanding of the scope and impact of this phenomenon and how it can influence decision-making. Furthermore, we suggest different mitigation strategies, support, and training of HCWs and school staff regarding manifestations and treatment of post–COVID-19 condition in children and young people across all demographic areas.

## Appendix

Multimedia Appendix 1 Filters used for the search strategy on Twitter.

Multimedia Appendix 2 Sentiment analysis framework: attitudes toward post–COVID-19 condition in children and young people.

Multimedia Appendix 3 Theme codebook: examples of tweets that fit into main themes tagged for mention of children and young people with post–COVID-19 condition.

Multimedia Appendix 4 Timeline of national governmental policies and guidelines regarding children and young people.

## Abbreviations

HCW: health care worker
GP: general practitioner
LISTEN: Collaborative and Digital Analysis of Big Qualitative Data in Time Sensitive Contexts
