# Supplementary material for: Twitter Analysis of Health Care Workers’ Sentiment and Discourse Regarding Post–COVID-19 Condition in Children and Young People: Mixed Methods Study
Source: J Med Internet Res. 2024 Apr 17;26:e50139. doi: 10.2196/50139 (PMC11063881; doi:10.2196/50139)
Supplement: Multimedia Appendix 1 [file jmir_v26i1e50139_app1.docx]

## Appendix 1

**Filters used for the search strategy on Twitter.**

The boolean string provided (see Table 1.) is designed to capture a wide array of discussions related to Long COVID or Post-COVID conditions, specifically targeting the terminology used, symptoms experienced, the affected demographic (children and young people), and the professionals involved in care (healthcare workers). Here is the rationale for the inclusions and exclusions within the context of the main paper:

1. **Terminology Inclusion ("Long Covid" OR "Post-COVID Conditions" OR "PASC" OR "long haulers"):** These terms were included to capture the broad spectrum of ways people refer to the condition post-COVID-19 infection. Including different terminologies ensures comprehensive coverage of the topic, as different regions, studies, or individuals may prefer one term over another.
2. ***Symptom Inclusion (symptom OR* " *chronic* condition*" OR fatigue...):**** A comprehensive list of symptoms was included to cover the wide range of Long COVID presentations, as reported in the literature and patient testimonies. This inclusivity is important as Long COVID is characterized by a wide range of symptoms that can affect multiple organ systems and can be described in various ways by patients. The use of wildcards (*), such as in symptom* or "chronic* condition*", ensures that variations of the words are covered (e.g., symptom, symptoms, symptomatic).
3. ***Demographic Inclusion (kid OR child* OR school*...):**** The boolean string includes terms that encompass all potential age groups of children and young people, which is relevant to the focus of the paper on Long COVID in a younger population. It ensures that conversations specifically about these groups are captured.
4. ***Professional Inclusion (nurs OR physio* OR physici*...):**** This part of the boolean string includes various healthcare worker roles to understand the professional discourse on Long COVID. It may also help in identifying information on how different healthcare professionals are discussing or reporting Long COVID in children and young people, reflecting the interdisciplinary nature of Long COVID management.
5. **Exclusion Criteria (NOT "lecturer" NOT "academic" NOT "professor"):** The exclusion of terms such as "lecturer", "academic" and "professor" may be designed to filter out discussions that are more theoretical or educational in nature, rather than clinical or experiential accounts of Long COVID. This helps to focus the search on practical, hands-on experiences and perspectives, which may be more relevant to the study's goals of understanding real-world impacts and management strategies.

The following boolean terms were crafted to support the objectives of the study by focusing on drawing out posts from social media accounts that shared patient experiences, symptomatology, and the responses of healthcare systems as they pertain to Long COVID in children and young people. It aimed to balance comprehensiveness with specificity to sample the most relevant and informative discussions captured from social listening platforms.

**Table S1**

| Variable | Descriptor |
| --- | --- |
| Long Covid keywords | ("Long Covid" OR "Post-COVID Conditions" OR "PASC" OR "longhaulers")  AND |
| Symptom keywords | (symptom* OR "chronic* condition*" OR fatigue OR "shortness of breath" OR "cough*" OR "chest pain" OR "joint pain" OR palpitations OR fever OR "loss of taste" OR "loss of smell" OR anosmia OR "difficulty concentrating" OR "brain fog" OR "headache*" OR dizziness OR depression OR anxiety OR "insomnia*" OR "extreme tiredness" OR "wiped out" OR "worn out" OR "breathless*" OR "can't breathe" OR "weird taste" OR "weird smell" OR "tingl*" OR "forgetful*" OR "can't think" OR "restless sleep" OR "up all night" OR "down in the dumps" OR "low mood" OR "worried all the time" OR "can't shake the worry" OR "heart flutter*" OR "irregular heartbeat" OR "runny tummy" OR "stomach ache*" OR "tummy trouble" OR "nausea*" OR vomiting OR "sore muscles" OR "aching joints" OR "feeling faint" OR "pins and needles" OR "numb*" OR "hair loss" OR "alopeci*” OR " taste*" OR "rash*" OR " covid toe*" OR "tachycardi*" OR "weak" OR "blurred vision" OR "hard to move" OR "struggling to remember" OR "cognitive issues" OR "mental fog" OR "stomach cramp*" OR "gut issue*" OR "lost pounds" OR "weight loss")  AND |
| CYP keywords | (kid* OR child* OR school* OR young* OR parent* OR toddler* OR nurser* OR kindergarten* OR boy* OR girl* OR teen* OR teenager* OR infant* OR minor* OR underage* OR "primary school" OR "secondary school" OR classroom* OR playground* OR adolescen* OR paediatric* OR pediatric* OR youth* OR "high school" OR "junior high" OR "elementary school" OR student* OR pupil*)  AND |
| HCW Bio keywords | ("nurs*" OR "physio*" OR "physici*" OR "midwife" OR "obstetrician" OR "geriatric*" OR "HCW" OR "health care worker" OR "healthcareworker" OR "doctor" OR "GP" OR "general practitioner" OR "health care professional" OR "health professional" OR "pharmac*" OR "radiograph*" OR "therap*" OR "neurologist" OR "psy*" OR "clinic*" OR "ambulance" OR "NHS" OR "osteopath" OR "orthoptist" OR "immunologist" OR "oncologist" OR "endocrinologist" OR "gastro*" OR "medical" OR "medic" OR "audiologist" OR "sonographer" OR "cardio*" OR "paediatric*" OR "pediatric*" OR "dietitian" OR "paramedic" OR "pathologist" OR "anaesth*" OR "anesth*" OR "orthoptist" OR "surg*" OR "dermatologist" OR "dentist" OR "geneticist" OR "rheumatologist" OR "urologist" OR "haematologist" OR "neonatologist" OR "intern*" OR "podiatry" OR "neurosurgeon" NOT "lecturer" NOT "academic" NOT "professor") |
