# Supplementary material for: Twitter Analysis of Health Care Workers’ Sentiment and Discourse Regarding Post–COVID-19 Condition in Children and Young People: Mixed Methods Study
Source: J Med Internet Res. 2024 Apr 17;26:e50139. doi: 10.2196/50139 (PMC11063881; doi:10.2196/50139)
Supplement: Multimedia Appendix 2 [file jmir_v26i1e50139_app2.docx]

## Appendix 2

### Sentiment analysis framework: Attitudes towards Long COVID in CYP

**Positive (P)**

- Post communicating overall trust and satisfaction with public health guidelines and support for treatment of Long COVID in CYP.
- Posts are affirming of official policies regarding the treatment of Long COVID in CYP.

**Negative (N)**

- Post contains negative attitudes/arguments against public health guidelines and support for treatment of Long COVID in CYP.
- Post discourages the following of recommended guidelines/support related to treatment of Long COVID in CYP - (for personal, political or other reasons).
- Post shares bad experiences of treatment of Long COVID in CYP.
