# Supplementary material for: Twitter Analysis of Health Care Workers’ Sentiment and Discourse Regarding Post–COVID-19 Condition in Children and Young People: Mixed Methods Study
Source: J Med Internet Res. 2024 Apr 17;26:e50139. doi: 10.2196/50139 (PMC11063881; doi:10.2196/50139)
Supplement: Multimedia Appendix 3 [file jmir_v26i1e50139_app3.docx]

### Appendix 3

### Theme Codebook: examples of tweets that fit into main themes tagged for mention of CYP with Long COVID

| Tag | When to apply | Example | Classification of the example according to the type of feeling |
| --- | --- | --- | --- |
| LCK – call for advice | Request advice on how to deal with LCK (symptoms, consultations, access to services, etc.) | Searching for a private practitioner to assess suspected brain disease in a child who already had COVID, London based, but telemedicine would be fine. Does anyone have any suggestions? #longcovid | Negative  ☹ |
| LCK – call for more support | Request more support from formal agencies (NHS). | I'm looking for advice... my teenage son is feeling fatigued and has been missing a lot of school. I think he has long covid I have received no support from the NHS. Any ideas? | Negative  ☹ |
| LCK- community support | Referred to expert support, support groups of parents, friends, or relatives, among others. | As a support group, we warmly welcomed over 100 new families with long covid children and listen to their stories first-hand. We look forward to supporting and guiding them during this time. | Positive  ☺ |
| LCK-facemasks | Discuss use of face mask as a pandemic control | Masks have many benefits, they especially protect those who do not wear them in closed spaces. However, it is important that they are used properly and are of quality. Let's worry about the children, we don’t know how many may get long covid! | Positive  ☺ |
| LCK- family concern | The comment alludes to their experience with LCK as a parent, familiar, friends, among others | I have a relative who is in school and has been diagnosed with covid for the second time. I’m concerned that parents are exposed to infection through their kids and with poor health and long covid. This government blatantly disregards this nation’s health | Negative  ☹ |
| LCK – expert opinion | Refers to an expert opinion, whether in a tweet, talk, lecture or otherwise | It’s been a fantastic day sharing my experience managing Long Covid in children at the Long Covid conference. | Positive  ☺ |
| LCK-health access | Refers to access to and use of health services (diagnosis, treatment, follow-up, acceptability, etc.) | We have a limited number of doctors and other health professionals in the NHS, not accounting for vacancies due to Long Covid. Workers do not multiply by magic! | Negative  ☹ |
| LCK – home organization | Discuss changes in household organisation due to covid | It has been difficult for me to deal with my job, raising my children and having long COVID. I feel like I need to distract myself somehow. | Negative  ☹ |
| LCK-images | A picture of a symptom, relevant event, health centre, among others, is shown. | Photograph of child and visual rash, with caption describing long covid fatigue, alongside the visual symptoms (image redacted for ethics) | Negative  ☹ |
| LCK – is real | Activism about the existence of Long Covid in kids, and rejection of denialism or minimisation of it | Do you know @Username? Children not only die from Covid, but also have prolonged long covid symptoms. | Negative  ☹ |
| LCK-mental health | Alludes to impact on mental health (distress, sadness, stress, depression, uncertainty). | Group Post I feel like I'm breaking down and unable to help my teenage daughter with long-term COVID. She has thoughts of self-harm and feels empty. It no longer supports this condition. He didn't want to tell me, so as not to cause concern. | Negative  ☹ |
| LCK- need for HW training | Need for education or further training of health workers for different long covid treatment/therapies | During my training as a doctor, I wasn’t taught that #GET (Graded Exercise Therapy) might not be helpful. What I’ve learned is only through my own illness with Long COVID. It is important to support/train future practioners and not demonise them. | Negative  ☹ |
| LCK – policy | Refers to formally issued directives or guidelines. | In recent weeks, the magnitude of infections in children under 15 years of age has reached more than 10 thousand cases... this is truly a crisis. I am concerned about the government's approach to this situation and the impact of long covid. | Negative  ☹ |
| LCK -research | Call to generate or disseminate more research in long covid | It is necessary to collect paediatric information in a public and open way to promote studies around the world, in a collaborative and independent way. #LongCovid #LongCovidKids | Neutral |
| LCK-School | Tells of LCK’s experience in schools (contagion, non-attendance) | I think it is necessary to learn to live with long covid, accepting that children will have it and may have prolonged symptoms or even die, considering that adolescents don’t get a vaccine booster, some children are not vaccinated, and schools do not overtly protect them. | Negative  ☹ |
| LCK- scientific article data | Publications or data from scientific articles or other formal studies are shared. | Government figures indicate that about 8% of infected children suffer from long covid. There is much uncertainty regarding the impact of this condition in the future. | Negative  ☹ |
| LCK - symptons | Describes presence of symptoms attributable to LCK | Group Post My school-age son has yet to be diagnosed with long covid, even though there is no cause for his prolonged stomach problems, fatigue, and low spirits #livingwithcovid | Negative  ☹ |
| LCK – treatment | Describes the use of any strategies to treat symptoms of long covid in children | There are organizations that have gained experience in managing Long covid in children and adults. These groups have gathered lessons and learning that they want to share with the community. | Positive  ☺ |
| LCK – uncertainty | Refers to uncertainty regarding symptoms, diagnosis, prognosis. | Does anyone know how frequently are children diagnosed with long covid? | Neutral |
| LCK-vaccine | Call or concern about vaccination in children and adolescents | Vaccines have proven to be effective. Considering the number of dead children and long covid cases, it is important to get vaccinated and wear masks. | Negative  ☹ |
| LCK -videos | A video of a symptom, relevant event, expert opinion, among others, is shown. | - | - |

## 
