# Supplementary material for: Twitter Analysis of Health Care Workers’ Sentiment and Discourse Regarding Post–COVID-19 Condition in Children and Young People: Mixed Methods Study
Source: J Med Internet Res. 2024 Apr 17;26:e50139. doi: 10.2196/50139 (PMC11063881; doi:10.2196/50139)
Supplement: Multimedia Appendix 4 [file jmir_v26i1e50139_app4.docx]

#### Appendix 4

## Timeline of national governmental policies/guidelines regarding CYP (Long Covid and schools). Sources: Official information from pages of the NHS [1], Gov.UK [2], and press [3].

| **Date of (first) publication** | **Name / Title** | **Source** | **Target Audience** |
| --- | --- | --- | --- |
| 27/04/2020 | COVID-19: paediatric surveillance | PHE | Healthcare Providers |
| 01/2021 | Schools’ closures are announced |  |  |
| 03/2021 | Reopening of school |  |  |
| 03/2021 | Face coverings are required in schools and colleges |  |  |
| 04/2021 | Face coverings no longer required in schools and colleges |  |  |
| 05/06/2021 | Long COVID plan 2021/2022: 10 key next steps to support those suffering from Long COVID | NHS | Healthcare Providers |
| 09/2021 | COVID-19 vaccination programme extended to 12-15 years old |  |  |
| 02/09/2021 | First findings from world’s largest study on Long COVID in children and young people (cites the: Non-hospitalised Children & young people (CYP) with Long COVID (The CLoCk Study)) | NIHR | Healthcare providers |
| 11/2021 | Face coverings are required in schools and colleges |  |  |
| 12/2021 | School closures are announced |  |  |
| 01/2022 | Reopening of schools |  |  |
| 01/2022 | Face coverings no longer required in schools and colleges |  |  |
| 02/2022 | End of mask-wearing policy |  |  |
| 02/2022 | COVID-19 vaccination programme extended to 5-11 years old |  |  |

REFERENCES:

1. NHS. Coronavirus » COVID-19 vaccination programme. NHS England and NHS Improvement coronavirus. Available from: https://www.england.nhs.uk/coronavirus/covid-19-vaccination-programme/ [accessed Mar 2, 2022]

2. GOV.UK. Actions for schools during the coronavirus outbreak. Guidance. Available from: https://www.gov.uk/government/publications/actions-for-schools-during-the-coronavirus-outbreak#full-publication-update-history [accessed Mar 2, 2022]

3. The Guardian. Boris Johnson tells schools in England to end mask-wearing policy. News. 2022. Available from: https://www.theguardian.com/education/2022/jan/21/boris-johnson-tells-schools-england-end-mask-wearing-policy [accessed Mar 2, 2022]
